# Inversion of the Uterus

**Published:** 1856-05

**Authors:** J. T. Jameson

**Affiliations:** South Otselic, Otsego County, N. Y.


ART. III.—*Inversion of the Uterus.* By J. T. JAMESON, South Otselic, Otsego County, N. Y.

MR. EDITOR: I read with much interest the case of inversion of the uterus, by Prof. White, as recorded in the March number of your Journal, and as the mode of reduction adopted by the Professor differs, not from my own experience, for I have had none in such accidents, but from the doctrine of that eminent obstetrician, Dr. Radford, of Manchester, England, a small volume of whose Essays on different subjects in midwifery I have in my possession, I concluded that it might be interesting and, perhaps, profitable to your readers, to communicate the point of difference. In his essay on the Inversion of the Uterus, Dr. Radford writes as follows: "When the placenta is detached, our next object should be, to attempt the reduction of the general bulk of the tumor, by compressing it. We are indebted to Mr. C. White for this method. The plan recommended by some writers, to push the fundus directly upward, should not be adopted. There are strong reasons to think that the fundus is, after the os uteri, the most irritable part of the organ. When the accident has existed a short time, pressure upon this portion induces pain, bearing down, and hæmorrhage; but the body may be taken hold of and compressed. *If we could press the fundus upward, and thereby dimple it within itself, we should find ourselves opposed by a double inflexion, for the body would be grasped by the os uteri, and the fundus would be within the body. It is obvious that our force should be directed so as to act upon the angle of inflexion, or where it turns into itself.*"

Now it will be seen from a reference to the practice of Prof. White, in the case above alluded to, that his modus operandi, so far as it regards the dimpling and pushing up the fundus, was in direct contrariety to the teaching of the English accoucheur. The reasoning of Dr. Radford appears good, but how far it will bear the test of actual practice, I am unable, from personal experience, to decide. I should feel obliged if Prof. White, or yourself, would see good to give us *the law* upon this important point in the treatment of such alarming and distressing cases.

Yours respectfully,

SOUTH OTSELIC, Otsego Co., N. Y. J. T. JAMESON.

*Editor's Comments.*—Our correspondent will find, on reference to Prof. White's case, that the first step in reposition is described by him as follows: "Introducing the entire right hand into the vagina, the whole body and fundus of the organ were firmly and continually compressed for some time."

The object of this was, of course, to relieve the engorgement, and diminish the bulk of the uterus. But we think we may say, without hesitation, for Prof. White, that this was his only object, and not in the language of Radford, "to attempt the reduction of the general bulk of the tumor, by compressing it." The reason is obvious to any person who has met the accident. Radford's plan is impracticable, and as to the "pain, bearing down, and hæmorrhage," the two first are incident to any manipulation, while the third is something that does not happen, so far as we are informed.

We are not aware of those "strong reasons," mentioned by Radford, for supposing the fundus the most irritable part of the organ—at any rate, *when in this condition.* In our own case (occurring immediately after labor) the patient made little outcry, and we do not think that it was more painful than many other obstetric operations. In Prof. White's case, the firm contraction incident to the long period which had elapsed, rendered the manipulation very painful, even under partial anæsthesia, but the shock from it was not great, there was not the least hæmorrhage, and no subsequent metritis. Finally, so far as Radford's plan is concerned, we believe it positively impracticable for the following reasons:

We introduce here a few words from our article on the subject, published in this Journal in Nov. 1853.

"First returning the uterus within the vagina, it should be carried bodily up until the vagina is 'on the stretch.' At this point it has been advised by Mr. Newnham, to 'return first, that portion of the organ which was last excluded from the os.' (Identical with Radford's plan.) How this is to be done in *complete* inversion, does not appear; in *partial* inversion it is possible, and is, I opine, the usual method of *completing* all inversions."

Reducing Radford's proposition to its simplest terms, it is proposed to reduce the neck of the uterus first. How is the os to be dilated in this plan? In the usual operation a "dimple" is created in the fundus; this is gradually pushed within itself, until the continuous folding of the organ upon itself brings the fundus of the organ in contact with the contracted os. It represents now a cone; its apex gradually insinuating itself through, and dilating the os, from below, while the walls of the vagina, completely on the stretch, aid by assisting in dilatation and by counter-extension, against the upward force. We can conceive of no more feasible plan for the operation.

A far more important point in securing the success of an attempt at reposition, is in supporting the uterus by the left hand from above. In our own case, we quote from the article describing it, that "the open and fully dilated os uteri could be felt through the abdominal parietes over the pubis. Dr.

Flint engaging his fingers behind this, externally, I introduced my entire hand into the vagina and gradually carried the tumor up."

By this support from above, very great assistance was gained, but its advantages are best expressed in Prof. White's report, as follows:

"Whenever these progressive efforts were resumed, the left hand was placed over the uterine tumor, which could now be distinctly felt in the hypogastrium. By means of the counter-pressure above the pubis, a much greater degree of compression could be made on the depression in the fundus of the uterus, without lacerating its vaginal connections. At length the fingers of the left hand being pressed well down into the abdomen, seemed to fasten upon or hook over the anterior uterine lip, and aid in its reflexion over the organ."

Our correspondent will see that it is only *through the walls of the abdomen*, that reposition can be made from the os. In conclusion, we may say that we are glad to have had the opportunity to defend the procedure referred to, regarding it, as we do, as the only practical one. Prof. White's case shows that it is also practicable in cases offering the most formidable obstacles.

